# The Need for a Systems Biology Approach in Cancer Explained

**DOI:** 10.3390/ijms27010141

**Published:** 2025-12-22

**Authors:** Hehuan Zhu, Xi Zhang, Ehsan Nazemalhosseini-Mojarad, Jessica Roelands, Lysanne D. A. N. de Muynck, Cor J. Ravensbergen, Rachel Hoorntje, Imke Stouten, Marianne Hokland, Alexander L. Vahrmeijer, Rob A. E. M. Tollenaar, Edwin Koster, Peter J. K. Kuppen

**Affiliations:** 1 Department of Surgery, Leiden University Medical Centre, 2333 ZA Leiden, The Netherlands; h.zhu@lumc.nl (H.Z.); x.zhang1@lumc.nl (X.Z.); i.stouten@lumc.nl (I.S.); a.l.vahrmeijer@lumc.nl (A.L.V.); r.a.e.m.tollenaar@lumc.nl (R.A.E.M.T.); 2 Department of Pathology, Leiden University Medical Centre, 2333 ZA Leiden, The Netherlands; j.p.roelands@lumc.nl (J.R.); c.j.ravensbergen@lumc.nl (C.J.R.); 3 Department of Biomedicine, Aarhus University, 8000 Aarhus, Denmark; mhokland@biomed.au.dk; 4 Department of Philosophy, Faculty of Social Sciences and Humanities, Vrije Universiteit Amsterdam, De Boelelaan 1105, 1081 HV Amsterdam, The Netherlands; e.koster@vu.nl

**Keywords:** cancer, systems biology, molecular biology, emergent property, upward causation, downward causation

## Abstract

Traditionally, scientists tend to approach cancer research in a reductionistic way: aiming at uncovering underlying, separate components in malignant processes. And indeed, great progress has been made by reducing the development of a tumor to single, specific genes and mutations. For instance, familial adenomatous polyposis (FAP) could be reduced to a germline mutation in the Adenomatous Polyposis Coli (APC) gene. The escape of tumor cells from immune surveillance could be reduced to the tumor expression of immune checkpoints, resulting in new approaches in tumor therapy by applying immune checkpoint inhibitors. However, a germline mutation in APC is not 1:1 related to colorectal cancer (CRC), and only some patients respond to immune checkpoint inhibitors. The point here is that biological systems, also comprising cancer, have properties that cannot be reduced to single components. The cooperation of the single components results in new, emergent properties. The outcome of an interaction in a complex network, like the immune system, depends on the many cell types involved and the numerous molecules that interact and activate or inhibit pathways. The way the composing elements are organized is a causal factor in itself for any emergent property. The rise of genomic analysis at the end of the previous century, enabling us to sequence a full genome at the DNA and RNA levels, has initiated an awareness of the need for ‘systems biology’: to consider a full system and how it is organized, in all of its aspects, to understand biological pathways and their outcomes. In this review, we outline the prospects and limitations of systems biology in cancer research and propose a causal framework that integrates upward and downward causation and multiple realizability to understand the emergent properties of tumors that determine the dynamics of tumor development.

## 1. Introduction

Usually, the focus in cancer research is reductionistic: searching for aberrancies in specific genes or proteins to explain tumor growth. The idea behind this is that basic components are responsible for tumor growth. Indeed, the composing elements at a lower organizational level affect properties at a higher organizational level. It may, however, also be an oversimplification, as properties at a higher organizational level cannot be simply reduced to the composing elements. The higher-level property is unique, emerging from the collaborative underlying elements due to the way they are structured and interact [1]. For example, when a T cell receptor is activated by a human leukocyte antigen (HLA) molecule that presents a virus-derived peptide, it triggers a cascade of interactions within the T cell [2]. These are all pure physical interactions between molecules, at a very basic level, and, in fact, between atoms. Physical laws, based on charge, mass, and more elementary properties, determine the outcome of such interactions between molecules and dictate the next interactive step [3,4]. At this level, it is nothing more than interacting molecules. At the level of an individual, however, the outcome is recovering from a viral infection. None of the involved molecules can be held responsible for recovering from a viral infection. The way the composing elements (molecules) are organized is a causal factor in itself for the emerging property [1], in this case, recovering from a viral infection. A similar example can, of course, be given for a T cell killing a cancer cell.

Cancer properties, such as uncontrolled cell growth, are also emergent properties, resulting from the way the composing elements are organized, and therefore, they cannot easily be reduced to mutations in genes or dysfunctional proteins. Genes are involved, and even responsible, but it is the interaction of the elements of the total system that results in the emergent property ‘uncontrolled cellular proliferation’. The way the composing elements are structured, including, for instance, the timing and level of protein expression, is crucial [5]. Typical for cancer cells is the expression of proteins that are normally not expressed or are expressed at a lower or higher level. This ruins the highly fine-tuned functional dynamics in a cell, resulting in ‘cancer’ properties: uncontrolled proliferation, infiltration into healthy tissue, and metastasis. ‘Systems biology’, comprising the dynamics of the full functional system, as will be explained, is needed to understand biological processes such as tumor development. This review, therefore, treats cancer as a multiscale system and uses upward and downward causation as a practical causal scaffold to organize mechanisms across levels, including immune and host constraints, to strengthen interpretation and guide systems-level research questions.

## 2. Causation in Biological Systems

**Figure 1:**
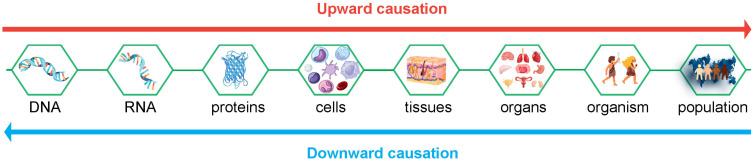
Representation of upward and downward causation in organisms. Each organizational level (boxes) is associated with specific emergent properties. Upward causation: Processes at a lower organizational level are responsible for processes at a higher organizational level. Downward causation is the other way around; processes at a higher organizational level cause effects on a lower level. In all cases, emergent properties cannot be reduced to the composing elements; the organization itself is a causative element. Note that the icons depict representative examples at each level rather than an exhaustive list, and the figure can be further extended on both sides.

As discussed above, the composing elements and the way they are organized are responsible for emergent properties observed at a higher organizational level. This is called upward causation. For instance, the process of apoptosis is initiated by a network of interacting proteins (the composing elements, lower organizational level) and results in cell death (emergent property, higher organizational level). Apoptosis in cells is a unique process that cannot be reduced to any of the composing elements; it is the elements and how they interact that cause the biological outcome: cell death, in this case. Upward causation underlies biology at every level, from DNA transcription and the resulting protein expression to the communication and cooperation among organs that together constitute a living organism. However, systems are not only characterized by upward causation. The opposite process also takes place: downward causation. Emergent properties are responsible for this downward causation: processes at a higher organizational level cause effects on a lower level. For instance, eating food activates the salivary gland to produce saliva: cells in the salivary gland respond to molecules from the food in their environment by activating signaling pathways, resulting in the production of digestive proteins and their secretion, together with fluid [6]. Therefore, a process at a higher organizational level (eating food) causes effects at a lower level (molecular processes that lead to the secretion of saliva). A simple representation of biological upward and downward causation is shown in Figure 1.

Emergent properties also include processes that check the integrity of the property and may activate downstream processes, enabling them to maintain the emergent property. For instance, cells have a system to check DNA integrity (cell, higher organizational level) and can activate proteins to replace mutated nucleotides (DNA repair, lower organizational level). As another example, dying cells at the top of epithelial tissues (tissue, higher organizational level) cause cell proliferation at the base of the epithelium (single cells, lower organizational level). Together, upward and downward causation maintain homeostasis: the balance between processes at each organizational level needed to keep an individual healthy. For instance, consider having a migraine (higher organizational level), which is caused by defective molecular processes (lower organizational level) [7]. Therefore, a migraine is due to upward causation. A person with a migraine may take medicine to feel better. The drugs will interfere with processes at a molecular level (lower organizational level). Therefore, when the drugs stop the migraine, this is caused by downward causation.

## 3. Causation in Cancer

**Figure 2:**
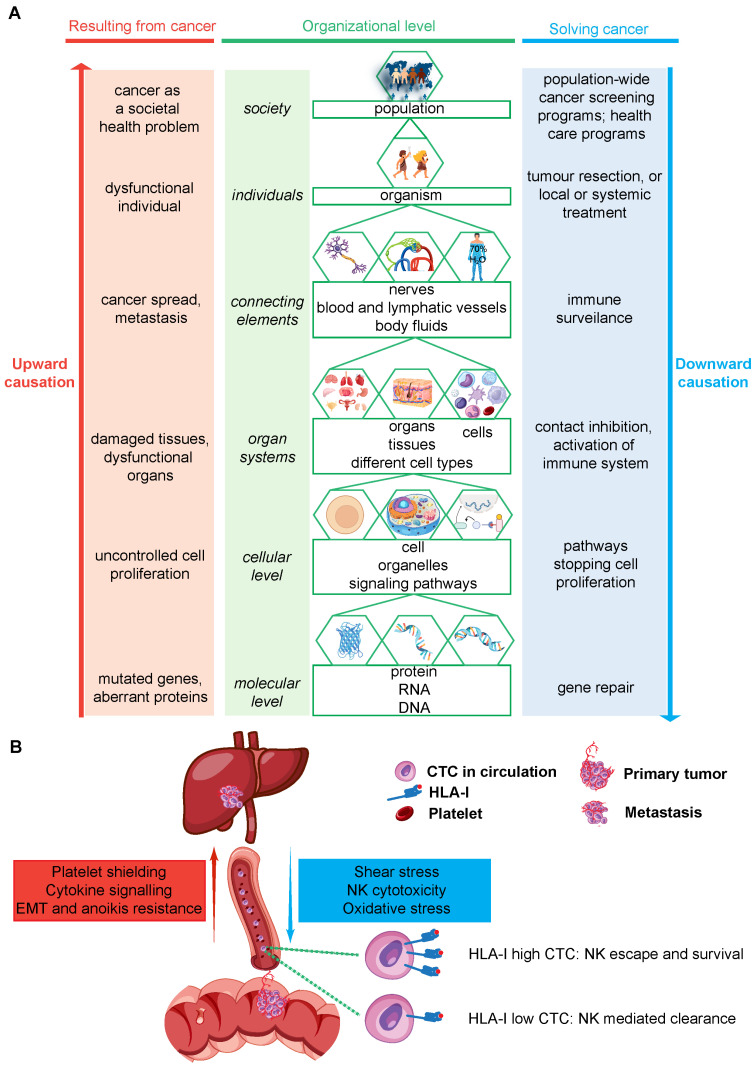
Upward and downward causation across organizational levels in cancer, with a zoomed view of circulating tumor cells. Panel (**A**) summarizes how emergent properties at the molecular, cellular, tissue, organ, organism, and population level can contribute to tumor development via upward causation and constrain tumor growth via downward causation. The listed processes are illustrative examples and do not represent all mechanisms operating at each level. Panel (**B**) expands the circulation level and depicts circulating tumor cells, escaped from a primary tumor in the intestine, as an interface for opposing forces. Upward enabling inputs include platelet shielding, cytokine signaling, and the induction of epithelial–mesenchymal transition with anoikis resistance, resulting in the growth of a metastasis in the liver. Downward constraints include shear stress, oxidative stress, and NK cytotoxicity, which may prevent the outgrowth of liver metastasis. Abbreviations: circulating tumor cells (CTC), epithelial mesenchymal transition (EMT), human leukocyte class I (HLA-I), natural killer cell (NK).

The development of cancer fully follows this model of biological causation (see, for instance, breast cancer [8]). In the case of tumor growth, it is both upward and downward causation that should be considered to understand the underlying processes. This is because it is not only processes that mistakenly induce cell proliferation but also failing correction processes that lead to tumor development. Cancer development affects emergent properties at many different levels: from molecular interactions to society. In Figure 2A, this is further explained and illustrated. Any aberrancy at a lower organizational level causes a problem at a higher organizational level; this affects an emergent property or induces a new property, for instance, ‘tumor growth’ in an individual, or a company with a problem due to a staff member diagnosed with cancer. At all organizational levels, there are downward-directed processes intended to control tumor development. Failure at all levels of these control processes, as shown in Figure 2A, ultimately results in cancer as a societal health problem.

Therefore, to understand cancer, it is essential to understand both the upward processes that cause the problems caused by cancer and the downward-directed processes that are activated to control cancer. For each process, it is important to consider it, in toto, a process that results in an emergent property that cannot be simply reduced to the composing elements. Systems biology is, in fact, the outcome of upward- and downward-causing processes. At the time a patient experiences complaints due to a growing tumor, this tumor has developed due to many upward-causing processes and has already escaped from many processes of downward-directed control. For effective therapeutic intervention in this tumor development, a thorough understanding of both the causative processes and failing control mechanisms is critical. This framework of upward and downward causation, as shown in Figure 2A, will be further discussed below for each organizational level.

## 4. Molecular Level

At this level, molecules like DNA, RNA, proteins, and many more interact. The development of cancer is a multi-causal process. Approaches at this level include protein–protein interaction and signaling network modeling, pathway activity inference from bulk RNA sequencing, and genome-scale regulatory network reconstruction to link mutations to network perturbation [9,10,11,12]. At the base are mutations in genes, resulting from exogenous or endogenous factors, or a combination. Mutated genes result in aberrant proteins that do not function properly. DNA mutations can also be responsible for deregulated miRNA networks and epigenetic machinery that turns specific genes on and off, all resulting in the deregulated expression of proteins [13]. Proteins themselves can be defective due to a DNA mutation or can be expressed at too high or too low a level or at the wrong time due to DNA mutations elsewhere. In all these cases, the cooperation of proteins in networks is affected, causing insufficiencies in the emergent functional property associated with the network. DNA mutations caused by endogenous factors are probably mainly associated with errors in DNA sequences during DNA duplication in proliferating cells. This may explain why carcinomas are the most prevalent tumors, as they originate from epithelium, a tissue with a very high proliferative capacity. Exogenous factors causing mutations in genes are environmental factors, including chemical, physical, and biological factors.

Chemical factors: For many kinds of chemical substances, it is known that they are associated with cancer. For instance, tobacco smoke (risk of lung cancer, but in fact all kinds of cancer) [14]; asbestos (risk of lung cancer, mesothelioma) [15]; alcohol (risk of many cancer types) [16]; polyaromatic hydrocarbons in burned organic material like barbequed meat (risk of cancers in skin, lung, and bladder) [17]. Many more can be added to this list. Exposure takes place in the working environment, via nutrition, environmental pollution, consumer goods, or because of a lifestyle.

Evident physical factors that cause cancer are ionizing radiation and UV light. Both types of radiation carry enough energy to damage DNA directly, leading to mutations that can initiate cancer. UV light (from sunlight or tanning beds) is associated with skin cancer [18]. Ionizing radiation (from the environment or used for medical imaging purposes) may lead to skin cancer and many other types, like leukemia [19], and it may also induce secondary cancers after radiotherapy of a primary tumor [20].

The third class is biological factors. The microbiome is clearly associated with cancer, including viruses, bacteria, and parasites [21]. For instance, human papillomavirus is associated with cervical cancer [22]. Also, bacteria are currently getting a lot of attention for their association with cancer. A clear example is the involvement of Helicobacter pylori in gastric cancer [23]. For the chemical and physical factors, it is generally accepted that causing DNA mutations is at the base of inducing associated cancers. For the microbiome, this is less clear and a topic of research [12,21]. For viruses, integration in the genome or affecting the genome in another way may be an option. For bacteria and parasites, the causal relationship with cancer is not yet known. It is possible that these affect cancer development at a higher organizational level than the genome, for instance, by affecting tumor immune surveillance.

DNA mutations are inextricably linked with life. Therefore, it is very logical that evolution has resulted in an extremely sophisticated and diverse system for DNA repair [24]. This check for DNA integrity is at a higher organizational (cellular) level, where processes are in place that can activate DNA repair. When successful, DNA mutations are repaired (downward causation); when these processes fail, cellular function is disturbed, and, for instance, uncontrolled cell growth may be initiated (upward causation). This check for DNA mutations and DNA repair is also dependent on proteins that are coded for by the DNA and, therefore, also prone to mutations. Indeed, for several cancer types, it is obvious that the DNA repair system is affected [25]. For instance, in subtypes of colorectal cancer (CRC), it is the microsatellite DNA repair system that is dysfunctional [26].

## 5. Cellular Level

At the cellular level, the different cell types function as the basic living elements of an organism. Approaches at this level include single-cell RNA sequencing and single-cell multi-omics to resolve tumor cell states, trajectory, and lineage inference, as well as mechanistic or probabilistic cell state models to capture phenotype plasticity [27,28]. The genomic status of a cell, i.e., the DNA sequence and, among other things, epigenetic status of promoter methylation and histone modifications, determines which genes will be transcribed in that cell. All components together function in a highly organized manner, enabling a cell to function.

The development of a tumor is a complex process. It is typically an outcome of multiple realizable processes: many roads can lead to cancer. At the base are DNA mutations that affect molecular pathways, which are networks that control cellular functions [29]. Via many different routes, the result can be uncontrolled cellular proliferation. Defects in repairing DNA mutations are probably at the base of tumor development (upward causation). During tumor cell division, DNA mutations keep accumulating, which explains why a tumor is usually composed of cells with a heterogeneous genotype and phenotype. Next to DNA repair, many other control mechanisms in place (downward causation), like apoptosis, are defective, ultimately resulting in uncontrolled cellular division. The result is a continuum of tumor cell phenotypes, of which those that possess a selective growth advantage grow out.

The exponential growth in the development of omics techniques, especially single-cell DNA and RNA techniques, has extensively improved our knowledge of inter- and intra-tumor heterogeneity [30,31,32]. By incorporating profound knowledge of tumor cell heterogeneity in terms of DNA mutations, expression level of proteins, cell cycle stage, etc., into comprehensive computational models, systems biology enables a more holistic understanding of the complete tumorigenic process at the cellular level.

## 6. Organ Systems

Organs are composed of well-organized specialized tissues that contain different cell types that all together are responsible for the functioning of that organ [33]. Approaches at this level include spatial transcriptomics and multiplexed imaging to quantify tissue architecture and cell–cell interactions, together with digital pathology and ecological modeling of tumor ecosystems [34,35,36]. Maintaining tissue integrity is crucial for optimal organ functioning. Contact inhibition is a process of downward causation used to maintain tissue integrity and stop unnecessary cell proliferation. Especially for epithelial tissues, for instance, in ducts, which are constantly renewing tissues, this is an important process. If the status of a cell enables uncontrolled proliferation, and contact inhibition is also not functioning, a tumor starts growing and may infiltrate surrounding tissue [37]. This will initiate tissue damage, resulting in the secretion of specific cytokines and other molecules, activating a new level of processes of downward causation in stopping tumor growth. The secreted molecules are necessary to initiate healing of damaged tissue and attract the immune system for immune surveillance [37,38]. Tumors like carcinomas show a very broad spectrum of behavior and morphology, varying from undifferentiated, hardly distinguishable tumors, such as those derived from epithelial tissue, to highly differentiated tumors, looking like epithelial tissue but growing inside the organ [39]. Surrounding tissue responds to this growing epithelial tissue, for instance, by inducing growth in supportive tissue, like that needed for the normal epithelium, which is visible as stromal tissue; together, this is often referred to as the tumor microenvironment [40]. For a systems biology approach at this level, it is important to understand both the processes in tumor cells and in the reacting normal cells.

## 7. Connecting Elements

The organs are connected via nerves, blood vessels, lymphatic vessels, and body fluids. Via this system, organs can communicate and respond to signals. Approaches at this level include longitudinal liquid biopsy profiling of circulating tumor cells and peripheral immune subsets, plasma proteomics, and dynamic network models that integrate soluble factors and immune surveillance across compartments [41,42,43]. Tissue damage signals coming from organs with a growing tumor may alert the immune system and attract immune cells to these sites. If tumor cells are sufficiently recognized by the immune system as aberrant, for instance, due to presentation of peptides derived from mutated proteins, these may be killed before becoming clinically evident [44]. However, with genetic instability as the driving force, tumor cells may arise that are able to escape from immune control and selectively grow out.

Except for growing into adjacent organs, tumor cells depend on vessels to migrate to distant organs [45]. A part of primary tumor cells may have acquired additional emergent properties needed to metastasize successfully and enter the blood or lymphatic vessels, potentially leading to distant metastases [46]. These tumor cells, then called circulating tumor cells (CTCs), arrive in a new, distinct location from the primary tumor. These opposing constraints and enabling signals in the circulation are schematized in Figure 2B. They are subjected to numerous restrictive barriers, which include immune surveillance, fluid shear stress, and increased oxygen tension, all downward-directed processes [47]. Next to restrictive barriers, the blood contains a wide range of soluble factors that can affect cell signaling pathways in CTCs. These factors comprise a complex signaling network and induce a plethora of dynamic signaling cascades in multiple cell types. For instance, the cellular activity of CTCs is closely related to multiple cytokines present in the circulation that activate signaling pathways involved in (via upward causation) migration, proliferation, epithelial–mesenchymal transition (EMT), and anoikis/apoptosis resistance [48,49,50]. Platelets are important producers of these cytokines [51]. However, these cytokines are also secreted by cells from the primary tumor, emphasizing the importance of a systems biology-based approach in cancer research, as CTC behavior is not solely mediated from within the circulation, but also by the primary tumor itself.

A selection process takes place, and while the vast majority of CTCs will not survive, some CTC clones are able to adapt and survive by modulating properties at a molecular level. Although these adaptive responses are still poorly understood, mRNA sequencing of CTCs from CRC patients that are exposed to continuous flow (mimicking shear forces) has demonstrated that several EMT-related genes are upregulated, together with the significant regulation of approximately 100 other genes [52]. These findings suggest that biomechanical forces induce a downward causation process, resulting in transcriptomic changes that, in turn, have an influence at the cellular level of CTCs: increased epithelial-plasticity and morphological changes. Thus, even though the stress factors present in the circulation greatly limit CTC survival, this also results in the emergence of CTC clones with increased metastatic potential, as they can better cope with mechanical and biochemical stress. In addition, many more genes are differentially expressed in the CTCs of CRC patients as compared to the primary tumor [53,54]. It has become evident that some CTC clones have acquired an aggressive phenotype, with an increased expression of genes involved in chemoresistance, apoptosis resistance, organ colonization, and decreased expression of tumor suppressor genes.

An important barrier for CTC may be the immune system. The interaction between tumor cells and immune cells will be very different in the circulation, as immune cell composition differs greatly from that of immune cells present in a tumor. For instance, in the primary tumor environment, NK cells are present at much lower frequencies than in the blood. [55]. CRC cells often show downregulation of classical HLA class I (HLA-I), resulting in immune escape from effector T cells [56,57,58]. However, in the circulation, NK cells comprise 10–15% of the peripheral blood lymphocyte population [59,60,61]. In this context, metastatic tumor clones that lack HLA-I expression will be eliminated, while HLA-I-positive metastatic cells have a survival advantage. Indeed, it has been reported that partial downregulation of HLA-I in CRC has a worse prognosis than complete loss of HLA-I in CRC patients [62]. Interestingly, the cytotoxic and cytolytic activity of circulating NK cells seems to be impaired in CRC patients, suggesting that the downward-directed control process of immune surveillance for tumor cells in the circulation is overruled by downward-directed processes initiated by the tumor, resulting in impaired immune functioning [60,63].

Important for understanding tumor-immune interactions is the acknowledgement that tumor–immune interactions are inherently multivariate, operating across different time and spatial scales and cell types. Due to the evident successes of immunotherapy in some cancer types [64,65] and the technological and computational advances in life sciences [66,67], tumor immunology currently stands at the forefront of biomedical research. However, there is an urgent need to distinguish between patients who will respond to therapies and those who will not. Systems biology helps to address this question by integrating knowledge of the interactions between multiple cell types, receptors, and cytokines as they travel through different anatomical locations to organize an effective immune response. Studying CTC and peripheral immune subsets is particularly interesting, as blood is very easily drawn from a patient at multiple different time points. Analyzing the peripheral immune profile of patients adds yet another level to our tumor biology understanding, and investigating tumor–immune interactions at different levels in a patient (for instance, the primary tumor, circulation, and metastatic site) enables us to acquire a comprehensive idea of the immune system in relation to tumor progression and therapy response [60,61,68]. A systems biology approach will improve treatment options and enable better personalized care for patients with advanced tumors.

Taken together, once a tumor cell enters the circulation, it becomes exposed to a completely different environment with different selective constraints. Cancer metastasis is a multifaceted process, unfolding at many biological and anatomical scales. The molecular characterization of CTCs, also including epigenetic phenomena, is vital for deciphering the biological aspects of the metastatic cascade. An evident challenge is to transform the concept of individual events during metastasis into an integrative, multiscale metastatic framework. While important components governing metastasis have been slowly elucidated in a reductionist manner, all metastatic events and aspects of CTCs are, by some means, connected to each other, and it is inadequate to only analyze aspects individually. Systems biology that integrates expertise from different fields (such as cancer biology, genetics, mathematics, and bioinformatics) can unravel the organization of the full metastatic cascade and make useful predictions for therapy.

## 8. Individuals

Every organism has unique properties in physical and psychological appearance. Approaches at this level include integrated multi-omics for patient stratification, immune profiling, and predictive modeling of treatment responses using interpretable machine learning and causal inference frameworks [12,69]. Firstly, this distinction among individuals can be reduced to the lowest organizational level: polymorphisms present in the DNA have an effect via upward causation processes at a higher organizational level. Secondly, each organism experiences different environmental interactions. Signaling from the outer world (through food, education, training, etc.) affects, via downward causation, the transcription status of genes, leading to further distinction in physical and psychological features among individuals. Due to this process, for example, genetically identical twins become different individuals. These processes are also at the base of tumor development: a tumor is characterized by a certain DNA sequence and epigenetically regulated gene transcription in which the environment plays an important role. Therefore, a similar tumor in two individuals will always be different. This justifies the rise of personalized medicine to treat cancer. It was genomic studies that showed genetic and epigenetic variation, and this is what made people realize the importance of personalized medicine.

In addition, at the level of the individual, co-morbid conditions, such as diabetes, obesity, or chronic inflammatory diseases, interact with cancer development and progression through shared signaling pathways and systemic alterations, including metabolic and immune changes. These represent downward causative processes, where a disease at the organismal level affects molecular and cellular mechanisms that influence tumor growth and therapy response. Conversely, cancer itself may exacerbate co-morbid conditions, illustrating upward causation. Therefore, incorporating co-morbidity into systems biology models is essential for accurate prediction of prognosis and for tailoring personalized treatment strategies [70].

A tumor may be present for a long time before becoming clinically manifest. Once diagnosed, the best treatment option by far is surgical resection. For cancers that are already disseminated through the body, systemic treatment is preferred, perhaps in combination with surgery. The clinical outcome may differ greatly between individual cancer patients, even in cases of patients with seemingly similar starting points. This may be caused by large differences in the upward and downward causative processes that led to this cancer. Therefore, by considering these processes, for instance, through the molecular analysis of the tumor and its microenvironment at all the different levels considered and described here, a better personalized prediction of effective treatment and clinical outcomes can be made.

## 9. Society

The lifetime risk of developing cancer is around 50% [71,72]. Therefore, cancer has a very high impact on our society. The high incidence and early loss of life have initiated many initiatives to cope with cancer as a society, for instance, by raising funds to support cancer research to understand the development of the disease. Also, at the governmental level, initiatives are taken up to cope with cancer. All are focused on decreasing the incidence and mortality of cancer and improving the quality of life of cancer patients. These measures represent downward causation, where collective societal action influences the biological course of the disease through improved prevention, earlier detection, and better treatment. At the same time, biological processes, such as rising cancer incidence due to aging populations or lifestyle-related risk factors, exert upward causation by shaping societal priorities and policies.

**Figure 3:**
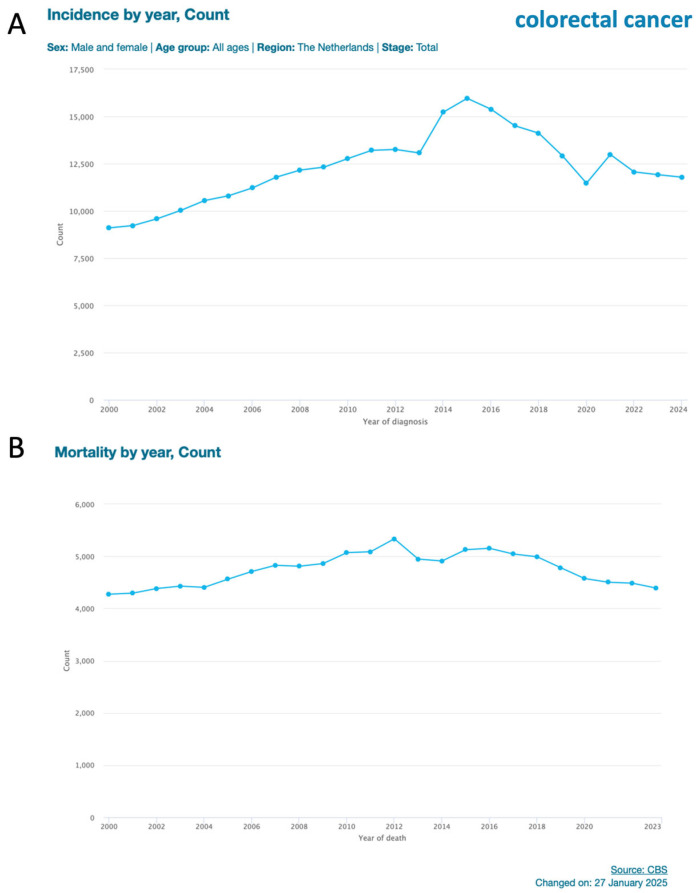
Incidence and mortality of colorectal cancer in the Netherlands [76]. Figures are from The Netherlands Cancer Registry [76]. (**A**) shows data on incidence (year 2000 to 2024) and (**B**) on mortality (years 2000 to 2023) of colorectal cancer. Population-wide screening based on a fecal occult blood test was introduced in 2014 in The Netherlands. The dip in 2012 in incidence is most likely due to the coronavirus pandemic at that time. Due to travel restrictions, fewer people with suspected cancer visited a hospital during the pandemic.

A clear example of downward causation at the societal level is the implementation of population-wide screening programs [73]. This is attractive as most cancers are very treatable, resulting in complete cures in the early stages. In the Netherlands, two population-wide screening programs are running for breast cancer [74] and CRC [75]. The program on CRC was stepwise introduced starting in 2014. People aged 55 to 75 years old are invited to send in a fecal sample to test for the presence of blood. Essentially, the program reduces CRC to hemoglobin present in feces. If hemoglobin is detected in the feces, a person will receive an invitation for a colonoscopy. As such, the program detects CRC at a much lower organizational level (leakage of blood) than that which an individual would detect (pain, fatigue, or other complaints). With this early detection, tumor progression is at an early stage and could have a higher chance of successful treatment. The question is whether this indeed happens. In Figure 3, the incidence and mortality of CRC for the Netherlands are shown for the years 2000–2024 [76].

Figure 3 shows a remarkable pattern. After the start of the screening program in 2014, there was a very clear peak in incidence. After this two-year peak, there is a steep decrease in incidence. An explanation could be that the screening program indeed detects early CRC. The cancers that would have been detected when clinically manifest are now detected about two years earlier. This is supported by the mortality graph (Figure 3B). This shows a gradual decrease in CRC mortality from 2016 onward. The pattern suggests that the screening works as intended: early-stage detection and less mortality. An argument against such screening programs, besides high costs, is that CRC patients are now identified in whom the cancer would never have become clinically manifest due to protective downward-directed processes such as immune surveillance. The program has not run for long enough to discuss this point based on the data, but it is highly relevant, as being classified as a cancer patient often equates to a high mental burden. This illustrates the complex interplay between upward and downward causation at the societal level: biological realities influence public health outcomes, and societal choices in turn shape biological trajectories.

## 10. Multiple Realizability at All Levels

It is important to understand that multiple realizability is operative at each organizational level. Multiple realizability means that a different process leads to the same result. For instance, many different pathways can lead to tumor development. In colorectal cancer, four main pathways are distinguished that are active in tumor development. Within these pathways, variations are possible, all with the same result: a tumor in the colon [77]. This is an example of the multiple realizability of upward causation. The same holds true for downward causation: many different processes can have the same result. For instance, a cure for cancer can not only be effected via immune surveillance but also via therapeutic intervention.

In fact, at each organizational level, many different processes of upward and downward causation have the same result. This is a main reason why a reductionistic approach, i.e., reducing a process to its composing elements, will not provide a real insight into the process. Only a systems biology approach, considering not only the composing elements but also the organizational structure and starting point and endpoint, can provide a comprehensive insight into the process and its dynamics, as will be explained below.

## 11. A Systems Biology Approach

Cancer is an exceedingly complicated illness, at all organizational levels, as discussed here. Efforts to profile tumors in the lab have shown a wide range of molecular and cellular changes, but their functional implications have yet to be fully discovered. Characterizing the genetics and epigenetics of cancer and the involvement of specific intracellular pathways in tumor initiation and development has come a long way [68]. The variety of hallmarks available for systems-level probing will continue to expand as the underlying cancer biology is better understood. The difficulties encountered when interpreting single-parameter research underline the need to understand cancer as a whole system. While integrative approaches are invaluable for uncovering fundamental cancer mechanisms [12,78], translating them into clinically actionable insights remains a challenge. For systems biology to meaningfully impact patient care, models must be interpretable and practical enough to inform diagnostics, prognosis, and treatment decisions in routine clinical practice. The rising success, as well as challenges, of cancer immunotherapy illustrates this concept well, where a simultaneous awareness of the communication networks within and across cell types in the tumor microenvironment will be critical for extending this treatment modality’s efficacy. The intricacy of cancer clearly necessitates a multi-pronged, multidisciplinary approach to understand the full systems biology involved in cancer development. Systems biology embraces the complexity of cancer by using massive multiscale data sets to construct integrative and predictive models that provide a systems-level knowledge of how molecular–cellular–organic changes interact to promote tumor growth.

One concrete route to make such models predictive is to use explicit in silico formalisms that translate system-level constraints into quantitative cell-level outputs, thereby operationalizing downward causation. For example, genome-scale metabolic network models combined with Flux Balance Analysis can implement organismal-or tissue-level nutrient and oxygen availability as constraints on cellular exchange fluxes, resulting in shifts in feasible intracellular flux distributions [79,80]. These constraint-driven flux reallocations can then propagate upward causation by shaping emergent phenotypes, such as proliferative capacity, redox homeostasis, and resistance to apoptosis, and can be compared with transcriptomic, proteomic, metabolomic, or isotope tracing data for validation. To address tumor microenvironment heterogeneity and cell-to-cell interactions, systems biology increasingly relies on spatially resolved and single-cell measurements to parameterize explicit interaction models. For example, agent-based and hybrid multiscale models can represent discrete cell states together with local cytokine, oxygen, and nutrient fields, allowing emergent patterns such as immune exclusion, suppressive niches, and spatially structured therapy resistance to arise from a defined cell status [81]. In parallel, graph-based frameworks and ligand–receptor-steered communication models can quantify intercellular signaling and neighborhood-dependent regulation, providing a mechanistic link between cell state composition, spatial organization, and tumor control [82,83]. Network-based models are also central to describing metabolic reprogramming as an emergent system-level property [84]. In particular, genome-scale metabolic networks and constraint-based modeling formalize how coordinated pathway-level flux redistribution emerges from network topology under microenvironmental constraints, and this can be extended to multicell settings to represent metabolic coupling, competition, and nutrient partitioning between tumor cells and immune or stromal cells [85,86,87]. This provides a systems-level explanation for metabolic phenotypes that cannot be assigned to single enzymes in isolation.

It is argued here that systems biology should comprise the full system, even up to the level of society. This is ambitious and currently impossible. However, by considering the separate processes of upward and downward causation and how they interact, it may be possible to obtain a better view of cancer development. In addition, the acknowledgement that the organization of the composing elements causes new, emergent properties, together with the multiple realizability of properties, is highly important. Together, this will lead to a better understanding and better treatment of cancer. Given the high costs of cancer therapy, it is essential to identify patients most likely to benefit and to avoid ineffective treatment. Systems biology provides predictive tools for patient selection and response prediction, supporting value-based care.

## 12. Conclusions

As discussed above, systems biology is a complex process of upward and downward operating processes. Therefore, considering cancer requires a systems biology approach to unravel causation, in which many organizational levels can be distinguished. By studying just separate components, at best, a correlation can be established, but no causative relationship. Currently, molecular research networks of interacting proteins and regulating RNAs are getting more and more attention, which is a very good development, but not sufficient yet, as the context in which they operate is usually much broader. Developments in omics technologies and computer hardware and software, including artificial intelligence, will help us to better appreciate cancer in a systems biology approach. As discussed, a systems biology approach requires a framework of upward and downward causation, in which emergent properties and multiple realizability are explicitly recognized across organizational levels. A genomic approach provides a concrete means to operationalize this framework by linking molecular variation and regulatory networks to higher-level phenotypes while simultaneously revealing how environmental-, physiological-, and population-level constraints shape genomic organization and function.

## Data Availability

The original data presented in the study are openly available at the Netherlands Cancer Registry (NCR), https://iknl.nl/en/NCR, accessed on 19 December 2025.

## Abbreviations

The following abbreviations are used in this manuscript:

APC	Adenomatous polyposis coli
CRC	Colorectal cancer
CTC	Circulating tumor cells
EMT	Epithelial–mesenchymal transition
FAP	Familial adenomatous polyposis
HLA	Human leukocyte antigen
NK	Natural Killer cell
